# Clinically significant sub-clonality for common drivers can be detected in 26% of *KRAS/EGFR* mutated lung adenocarcinomas

**DOI:** 10.18632/oncotarget.17399

**Published:** 2017-04-24

**Authors:** Einav Simon, Tova Bick, Shada Sarji, Talia Shentzer, Elad Prinz, Liza Yehiam, Edmond Sabo, Ofer Ben-Izhak, Dov Hershkovitz

**Affiliations:** ^1^ Institute of Pathology, Rambam Health Care Campus, Haifa, Israel; ^2^ Institute of Oncology, Rambam Health Care Campus, Haifa, Israel; ^3^ The Technion Integrated Cancer Center, B. Rappaport Faculty of Medicine, Technion-Israel Institute of Technology, Haifa, Israel; ^4^ Institute of Pathology, Tel-Aviv Sourasky Medical Center, Tel-Aviv, Israel; ^5^ Department of Pathology, Sackler Faculty of Medicine, Tel-Aviv University, Tel-Aviv, Israel

**Keywords:** sub-clonality, lung adenocarcinoma, KRAS, EGFR, evolution

## Abstract

Genetic sub-clonality has been described in multiple malignancies, however the presence of sub-clonality for major drivers in lung adenocarcinoma and its clinical significance is a subject under debate. Using molecular and morphometric approach, 347 lung adenocarcinoma samples were analyzed for *KRAS* and *EGFR* sub-clonality, which was further correlated with clinical and pathological variables.

*KRAS* and *EGFR* mutations were identified in 100 (29%) and 82 (23%) cases, respectively. One hundred and forty four *KRAS* or *EGFR* positive cases were also available for morphometric analysis, among which 37 (26%) were defined as sub-clonal. The presence of sub-clonality was associated with shorter survival time (p=0.02). Interestingly, cases with sub-clonality were also associated with earlier disease stage (89% vs 66% stage I disease in sub-clonal vs clonal cases, respectively, p=0.01) and less lymph node involvement (8% vs 25% in sub-clonal vs clonal cases, respectively, p=0.02). Our findings demonstrate the presence of sub-clonality for mutations in common drivers in lung adenocarcinoma and link it both to earlier disease stage and to poor survival. These findings are in line with the different evolutionary models that can present with genetic sub-clonality.

## INTRODUCTION

Cancer is a short term evolutionary process. Tumor cells acquiring cancer driver mutations give rise to sub-clonal populations that undergo Darwinian selection that can lead to clonal expansion or eradication [1, 2]. Two major models of cancer evolution have been described, linear and branched [1]. In linear evolution a clone acquiring a beneficial mutation undergoes expansion and eventually eliminates the ancestral clone, whereas in branched evolution, different sub-clones expand in parallel. Both models are compatible with the presence of sub-clonality, where some of the mutations are present only in a subset of the tumor cell population.

Sub-clonality has already been reported in multiple malignancies [3–6]. Specifically, in lung adenocarcinoma high throughput sequencing analysis of different tumor lesions in a small set of patients showed evidence of sub-clonality and branched evolution for driver events [7–9]. As for the common driver events (i.e. *EGFR* mutation and *ALK* rearrangement) the presence of sub-clonality in lung adenocarcinoma is under debate. Comparison between different lung nodules and between primary and metastatic lesions showed up to 30% discordance for *EGFR* mutation [10, 11] and 50% for ALK immunohistochemistry [12], in some reports. Additionally, even within the same lesion, multi-region sampling provided evidence for sub-clonality for *EGFR* mutation status [13, 14] and for *ALK* rearrangement [15]. In one study, *EGFR* sub-clonality was specifically linked to micropapillary histological variant [16]. On the other hand, an analysis of 862 cases with *EGFR* mutations did not find dual mutations and different areas of the tumor as well as paired primary and metastasis were concordant for the mutation [17].

Several studies in recent years have linked sub-clonality to poor prognosis and the development of treatment resistance [18–20]. This is presumably because sub-clonal populations acquire resistance to therapies via different and parallel mechanisms. In lung cancer, several studies reported that sub-clonal mutations were associated with worse prognosis [21] and shorter time to disease relapse [9]. Sub-clonality specific for *EGFR* was also associated with shorter progression free survival [22, 23] and the presence of sub-clonal resistance *EGFR* T790M mutation was also linked shorter time to progression [24]. On the other hand, analysis of a large cohort of lung adeno- and squamous cell carcinomas did not find an association between high sub-clonality and patients' survival [25].

The disagreement between the different studies with regard to the presence of sub-clonality in lung cancer and its clinical significance might be linked to differences in the methodologies for determining sub-clonality. Currently, the most widely used approach to determine sub-clonality is based on sampling different lesions or multi-region sampling within the same lesion. Using this approach sub-clonality is defined by the presence of a certain mutation only in a subset of the areas examined. One potential limitation of the multi-region sampling method is that it assumes spatial clustering of the different sub-clones and therefore might miss sub-clonality if the different sub-clones are inter-mixed [26]. We have developed a molecular-morphometric approach to determine sub-clonality, which can overcome this limitation, and applied it successfully to the study of sub-clonality in colon, pancreas and thyroid carcinomas [27–29]. In the present study we applied this approach to determine the presence of sub-clonality and its clinical significance in early stage lung cancer.

## RESULTS

Three hundred and forty seven cases were analyzed in the study. The average age at diagnosis was 70±10 and 42% of cases were females. Seventy five percent had stage I disease at diagnosis (Table 1). Molecular analysis identified *KRAS* mutation in 100 (29%) cases and *EGFR* mutations in 82 (23%) cases. Six cases (2%) had both *KRAS* and *EGFR* mutation. The most common *KRAS* mutation was c.34G>T and the most common *EGFR* mutation was exon 19 deletion (Supplementary Table 1).

**Table 1:** Clinical-pathological characteristics of the patients

Age	70±10
**Sex**	
** Female**	147 (42%)
** Male**	200 (58%)
**Smoking**	
** Yes**	226 (76%)
** No**	70 (24%)
**Location^1^**	
** LUL**	77 (23%)
** LLL**	58 (17%)
** RUL**	118 (35%)
** RML**	15 (4%)
** RLL**	72 (21%)
**Size (cm)**	2.8±2
**Stage^2^**	
** I**	255 (75%)
** II**	52 (15%)
** III**	35 (10%)
**Histology**	
** Acinar**	103 (29%)
** Solid**	93 (27%)
** Lepidic**	38 (11%)
** Papillary**	65 (19%)
** Micropapillary**	21 (6%)
** Mucinous**	24 (7%)
** Fetal**	3 (1%)
**Molecular**	
** KRAS**	100 (29%)
** EGFR**	82 (23%)
** KRAS&EGFR**	6 (2%)
** WT**	159 (46%)

LUL: left upper lobe; LLL: left lower lobe; RUL: right upper lobe; RML: right middle lobe; RLL: right lower lobe.

^1^ Seven cases not included in the location - 2 pleura, 5 involving multiple lobes.

^2^ Five cases with no stage data available.

Cases that were positive for *EGFR* mutation tended to be slightly older than wild type (WT) cases (72±9 vs 69±11 in the *EGFR* vs WT groups, respectively, p=0.04, Table 2). Smoking was more common in *KRAS* mutation positive cases and in wild-type (WT) compared to *EGFR* mutation positive cases (88% and 78% vs 56% in *KRAS*, WT and *EGFR*, respectively, p=0.0002). Additionally, solid histological variant was less common in *EGFR* mutation positive cases (12% in *EGFR* positive cases vs 27% and 34% in *KRAS* positive and WT cases, respectively, p=0.002), whereas mucinous histologic variant was more common in *KRAS* positive cases (16% vs 0% and 4% in *EGFR* positive and WT cases, respectively p=0.0003). Survival analysis showed increased survival in the *EGFR* mutation positive cases compared to the *KRAS* mutation positive and the WT groups (p=0.02, Supplementary Figure 1). No statistically significant difference was found between the different mutation groups with regard to sex, location of the lesion, tumor size or disease stage (Table 2).

**Table 2:** Clinical-pathological differences between the different mutation groups

	*KRAS*	*EGFR*	Wild-type	P-value
**Age**	70±10	72±9	69±11	0.04^1^
**Sex**				0.06
** Female**	37 (37%)	44 (54%)	60 (38%)	
** Male**	63 (63%)	38 (46%)	98 (62%)	
**Smoking**				0.0002^2^
** Yes**	81 (88%)	32 (56%)	109 (78%)	
** No**	11 (12%)	27 (44%)	30 (22%)	
**Location**				0.28
** LUL**	24 (24%)	23 (28%)	27 (18%)	
** LLL**	20 (20%)	14 (17%)	24 (16%)	
** RUL**	36 (37%)	26 (32%)	54 (35%)	
** RML**	3 (3%)	1 (1%)	10 (7%)	
** RLL**	16 (16%)	18 (22%)	37 (24%)	
**Size**	2.7±2.3	2.7±1.5	2.9±2	0.6
**Stage**				0.6
** I**	75 (76%)	57 (71%)	118 (76%)	
** II**	17 (17%)	14 (17%)	21 (13%)	
** III**	7 (7%)	10 (12%)	17 (11%)	
**Histology**				<0.0001
** Acinar**	26 (26%)	31 (38%)	43 (27%)	0.16
** Solid**	27 (27%)	10 (12%)	54 (34%)	0.002^2^
** Lepidic**	9 (9%)	13 (16%)	15 (9%)	0.15
** Papillary**	14 (14%)	20 (24%)	32 (20%)	0.25
** Micropapillary**	8 (8%)	6 (7%)	7 (4%)	0.46
** Mucinous**	16 (16%)	0 (0%)	7 (4%)	0.0003^3^
** Fetal**	0 (0%)	2 (2%)	1 (1%)	0.19

LUL: left upper lobe, LLL: left lower lobe; RUL: right upper lobe; RML: right middle lobe; RLL: right lower lobe; WT: wild-type.

^1^ *EGFR* vs WT.

^2^ *EGFR* vs *KRAS*/WT.

^3^ *EGFR* vs *KRAS* vs WT.

**Figure 1:**
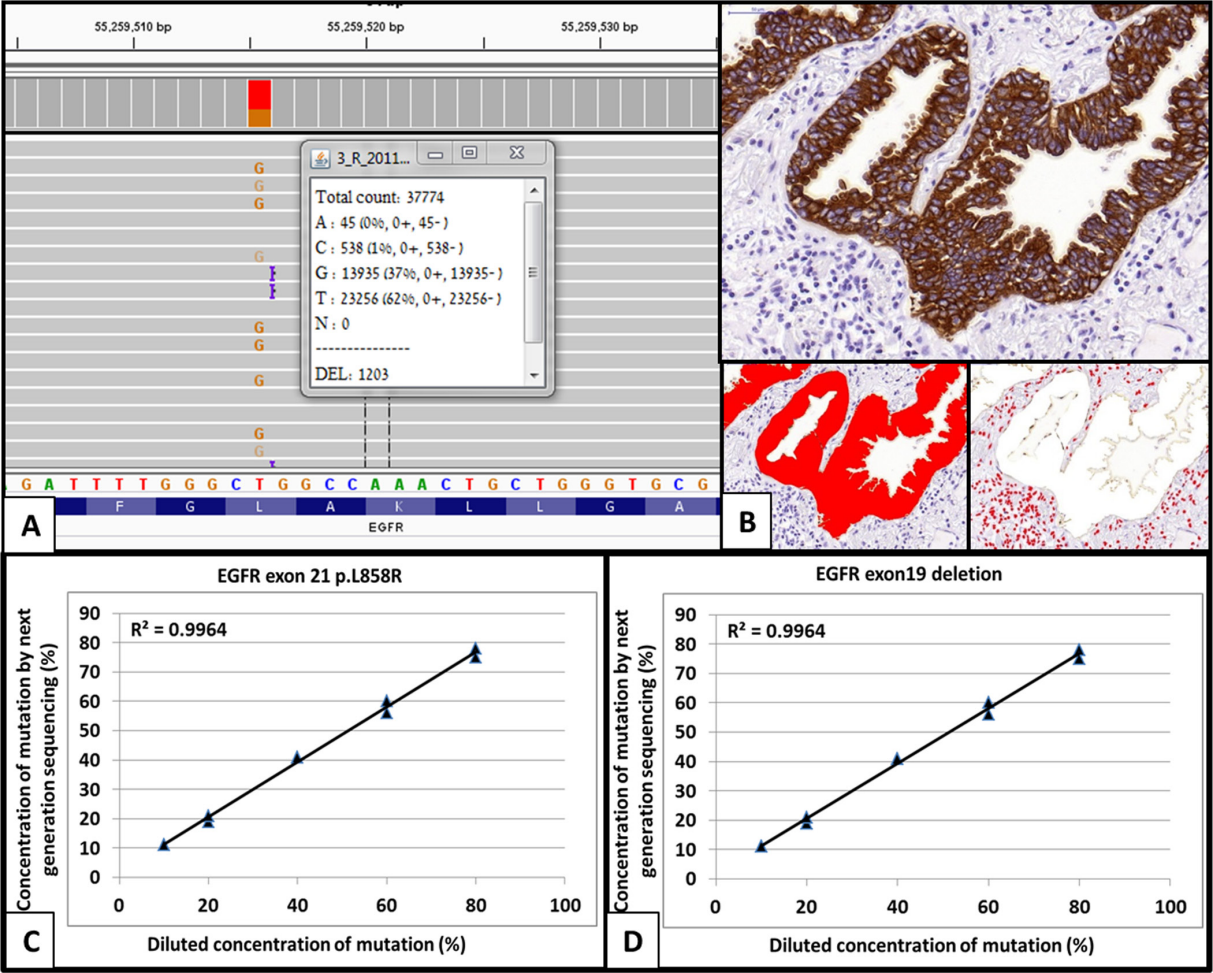
Molecular and morphometric analysis of sub-clonality for KRAS and EGFR mutations **(A)** A screen shot from the integrative genome browser software; the middle panel represents the DNA sequences the upper panel shows the summary of changes in each position and the lower panel is the reference genomic sequence. In this sample there is a specific T>G mutation (leading to EGFR p.L858R missense mutation). Using the program we were able to determine the mutation and fraction of mutant copies (inset). **(B)** For the morphometric analysis the slide was immunohistochemically stained with anti-cytokeratin antibody, and several representative high magnifications images were taken (upper panel). In each image tumor (left lower panel) and normal cell (right lower panel) numbers were counted using the Image Pro Plus program. **(C** and **D)** Standardized curve for validation of mutant fraction detection method. As detailed in the methods section, synthetic DNA harboring *EGFR* exon 21 **(C)** and exon 19 **(D)** mutations were diluted in wild-type synthetic DNA to concentrations ranging 10-80%. The different mixtures served as references for the standardized curve. Analysis of the data showed very high correlation between the predicted concentration of mutant *EGFR* and the concentration measured by next generation sequencing, confirming the reliability of our method. Using the morphometric and molecular results we were able to calculate the fraction of tumor cells harboring mutations and determine clonality status for the mutations.

Of the 188 mutation positive cases, 144 were available for both molecular and morphometric analysis. The average mutant allele frequency was 34%±19.7 (range 3-95%) and the average tumor cell fraction in the areas used for analysis was 44%±19.4 (range 3-88%). Bland-Altman analysis performed on repeated measurements of the morphometric analysis showed high agreement with percent difference up to 33% (Figure 1A). Due to the difference between repeated measurements and to avoid misclassification of cases as sub-clonal, only cases with a mutant allele frequency of less than 60% of expected, based on the morphometric analysis, were defined as sub-clonal. Additionally, cases with mutant allele frequency of more than 150% of expected were defined as mutant allele amplification. Following calculation of the fraction of tumor cells carrying a mutation 37 (26%) cases (31 cases with less than 60% of expected mutant allele fraction, 6 cases with double mutation) were defined as carrying sub-clonal mutation and 34 (23%) cases were defined as carrying mutant allele specific amplification (Figure 1B). Chromogenic *in-situ* hybridization with *EGFR* probe, performed on several cases, confirmed *EGFR* copy gains in cases that showed higher than expected mutant allele frequency (Figure 1C and 1D).

**Figure 2:**
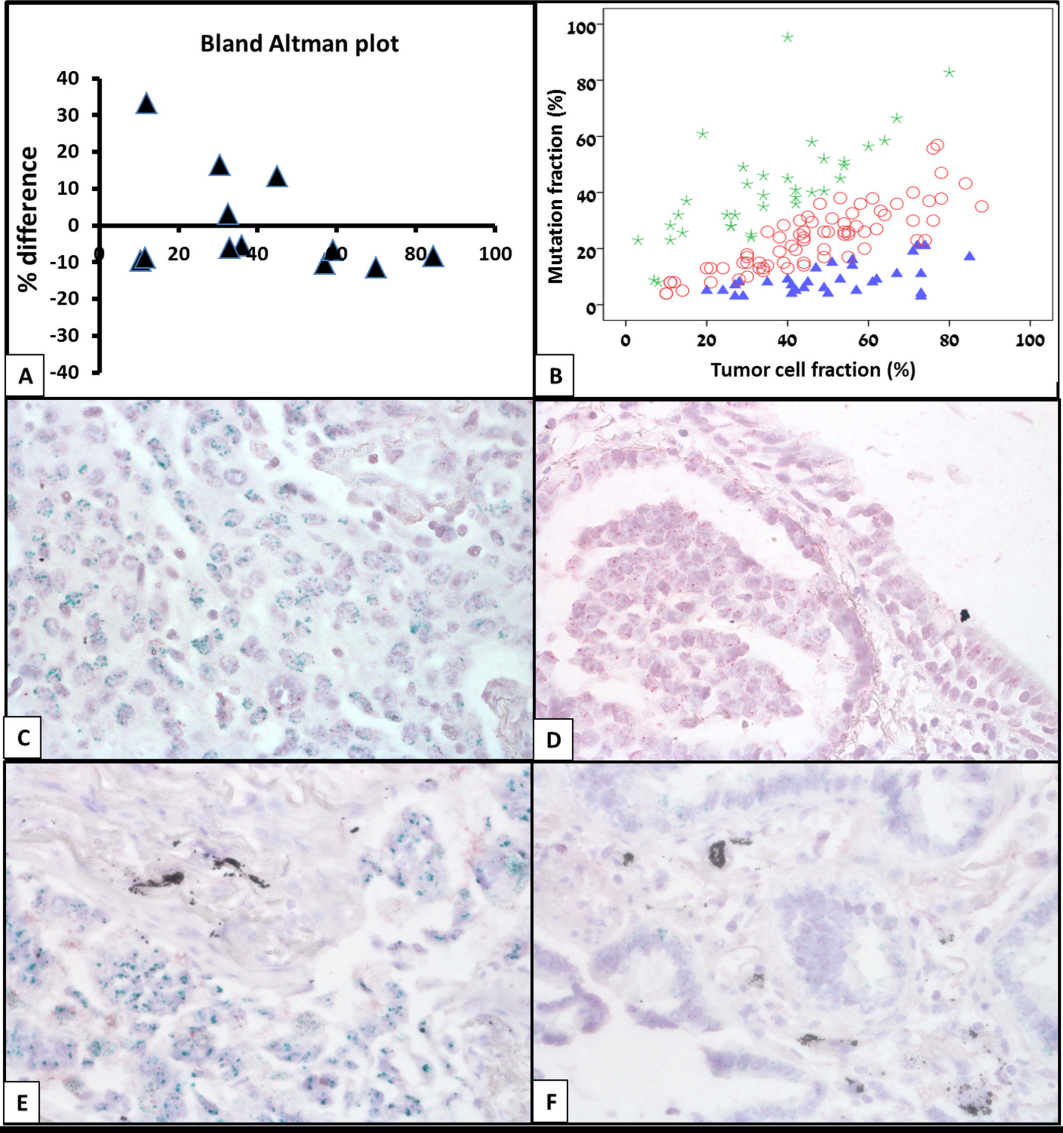
Identification of cases with KRAS or EGFR mutation sub-clonality **(A)** Bland-Altman analysis. To determine the reproducibility of the morphometric analysis the entire analysis process was re-performed on 12 cases. Bland-Altman analysis showed high agreement with maximal percent difference of 33%. To avoid misclassification of cases as sub-clonal, only cases that had mutant allele frequency of less than 60% of expected based on the morphometric analysis were defined as sub-clonal. **(B)** Based on the morphometric analysis, 37 cases were found to be sub-clonal (blue triangle) and 34 cases were found to harbor mutant allele specific amplification. **(C-F)** To validate the reliability of our molecular-morphometric approach several cases calculated to carry **(C, E)** and not carry **(D, F)** *EGFR* amplification underwent chromogenic *in-situ* hybridization (CISH) with anti *EGFR* probe. As demonstrated by CISH, multiple green dots in tumor nuclei, indicating *EGFR* amplification could be demonstrated in cases with predicted *EGFR* amplification **(C, E)** but not in cases with expected *EGFR* levels **(D, F)**.

**Figure 3:**
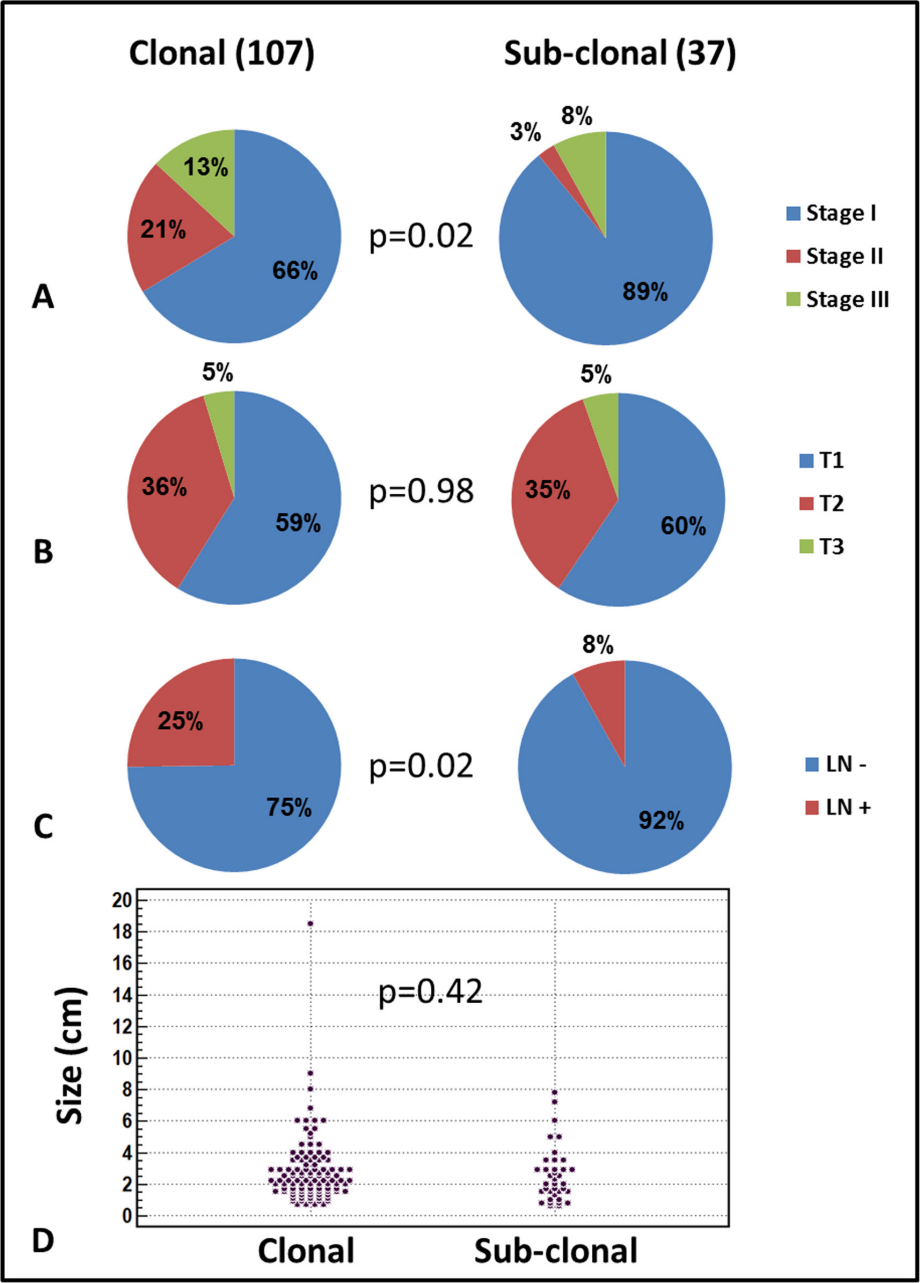
Clinical significance of *KRAS* or *EGFR* sub-clonality in lung adenocarcinoma Sub-clonality was associated with earlier disease stage **(A)**, and less lymph node (LN) involvemet **(C)**. No association was found between clonality status and tumor T stage **(B)** or diameter **(D)**.

Sub-clonal cases were associated with earlier disease stage (89% vs 66% stage I disease in sub-clonal vs clonal cases, respectively, p=0.01, Figure 2A) and less lymph node involvement (8% vs 25% in sub-clonal vs clonal cases, respectively, p=0.02, Figure 2C). No association was found between clonality status and tumor size (Figure 2B and 2D). Additionally, mucinous histologic subtype appeared at higher frequency in the cases with sub-clonal mutations (22% vs 7% in sub-clonal vs clonal cases, respectively, p=0.009, Table 3). Surprisingly, sub-clonality for *EGFR* mutation was associated with shorter survival time (p=0.03, Figure 3B) whereas sub-clonality for *KRAS* mutation only showed a trend toward shorter survival time (p=0.1, Figure 3A). The presence of sub-clonality for either *KRAS* or *EGFR* mutation status was associated with shorter survival time (p=0.02, Figure 3C). Cox multivariate regression analysis showed that disease stage and sub-clonality status were independent predictors of survival, and hence stage I disease with clonal mutation status had the best prognosis whereas cases with stage II or III disease with sub-clonality had the shortest survival time (p<0.05, Figure 3D). No association was found between the presence of mutant allele amplification and any of the clinical or pathological variables measured.

**Table 3:** Clinical-pathological differences between cases with and without sub-clonality

	sub-clonal	Clonal	P-value
**Age**	72±10	71±9	0.66
**Sex**			0.9
** Female**	18 (49%)	52 (49%)	
** Male**	19 (51%)	55 (51%)	
**Smoking**			0.6
** Yes**	24 (73%)	67 (77%)	
** No**	9 (27%)	20 (23%)	
**Size**	2.6±1.8	2.9±2.2	0.47
**LN involvement**	3 (8%)	27 (25%)	0.02
**Stage**			0.01^1^
** I**	33 (89%)	71 (66%)	
** II**	1 (3%)	22 (21%)	
** III**	3 (8%)	14 (13%)	
**Histology**			
** Acinar**	9 (24%)	39 (36%)	0.17
** Solid**	9 (24%)	21 (20%)	0.54
** Lepidic**	2 (5%)	14 (13%)	0.2
** Papillary**	8 (22%)	14 (13%)	0.21
** Micropapillary**	1 (3%)	11 (10%)	0.15
** Mucinous**	8 (22%)	7 (7%)	0.009
** Fetal**	0 (0%)	1 (1%)	0.5

LN – lymph nodes.

^1^ between stages I and II+III.

## DISCUSSION

Sub-clonality is an inevitable consequence of the dynamic nature of cancer progression. Tumor cells continuously acquire mutations, hence, at any given moment, at least those recently acquired mutation are sub-clonal. Supporting this concept, many research articles looking at single drivers or using comprehensive sequencing technologies were able to demonstrate sub-clonality in various malignancies [3, 30, 31]. There is also accumulating data suggesting that sub-clonality has clinical significance. The association between sub-clonality and treatment failure is biologically plausible. Small sub-clones with acquired resistance to therapy can undergo selection and cause disease progression [19]. Indeed, many studies found an association between sub-clonality and shorter time to disease progression in different malignancies such as chronic lymphocytic leukemia [32] and head and neck cancer [33]. Nevertheless, this subject is under debate with other studies linking sub-clonality to early disease stage for *BRAF* mutation in thyroid cancer [27, 34] and *KRAS* mutation in pancreatic [28] and colon cancer [35].

In the present study we found that sub-clonality for *KRAS* or *EGFR* mutation was associated with shorter survival times. The presence of sub-clonality for the common drivers in lung adenocarcinoma is in accordance with some previous reports [7, 8, 10, 15, 16]. However, different cohorts report lack of sub-clonality [17] or claim that it is probably a rare condition [36]. Interestingly, even among the studies that did identify sub-clonality, there is disagreement regarding its clinical significance with some associating sub-clonality with shorter time to progression [9, 22], whereas others report lack of clinical associations [11, 14, 25]. Moreover, low frequency *TP53* mutant allele fraction, interpreted as sub-clonality, was associated with better survival in cases of lung carcinoma [37].

One potential reason for the disagreement between different studies with regard to the clinical effect of sub-clonality might be related to the method used to measure sub-clonality. The most widely used approach to determine sub-clonality is based on comparison of different tumor lesions (e.g. primary tumor and metastasis) or multi-region sampling of a single lesion [4, 6, 7, 10, 11, 13, 15]. Using this approach, a tumor is defined as being heterogeneous if some mutations are present only in some of the samples. However, an intrinsic assumption of this approach is that different sub-clones are spatially separated, which might not be the case in many tumors. Interestingly, a recently reported model supports the rapid intra-tumor cell mixing during cancer evolution [38]. Considering the possibility of admixing of sub-clones, it is possible that multiple samples from different regions will be positive for a specific mutation despite it being present only in a subset of cells from each region. Mutant allele fraction in the entire sample being analyzed has also been evaluated as a tool to determine sub-clonality, with low allele frequency being defined as sub-clonality [39, 40]. The discordant results of these two studies looking into the clinical significance of sub-clonality for *BRAF* mutation in thyroid cancer might be the result of lack of correction for tumor cell fraction in each sample. Evaluation of the tumor cell content in the sample is essential to differentiate between true sub-clonality and low tumor cell content. A study linking high frequency *TP53* mutations with worse prognosis [37] is also limited by the lack of correction for tumor cell fraction. A molecular morphometric approach, like the one used in the present study should be able to accurately determine sub-clonality in intermixed tumor and also account for the tumor cell content in the sample. An alternative approach is to use laser microdissection and isolate only the tumor cells from the sample [41].

**Figure 4:**
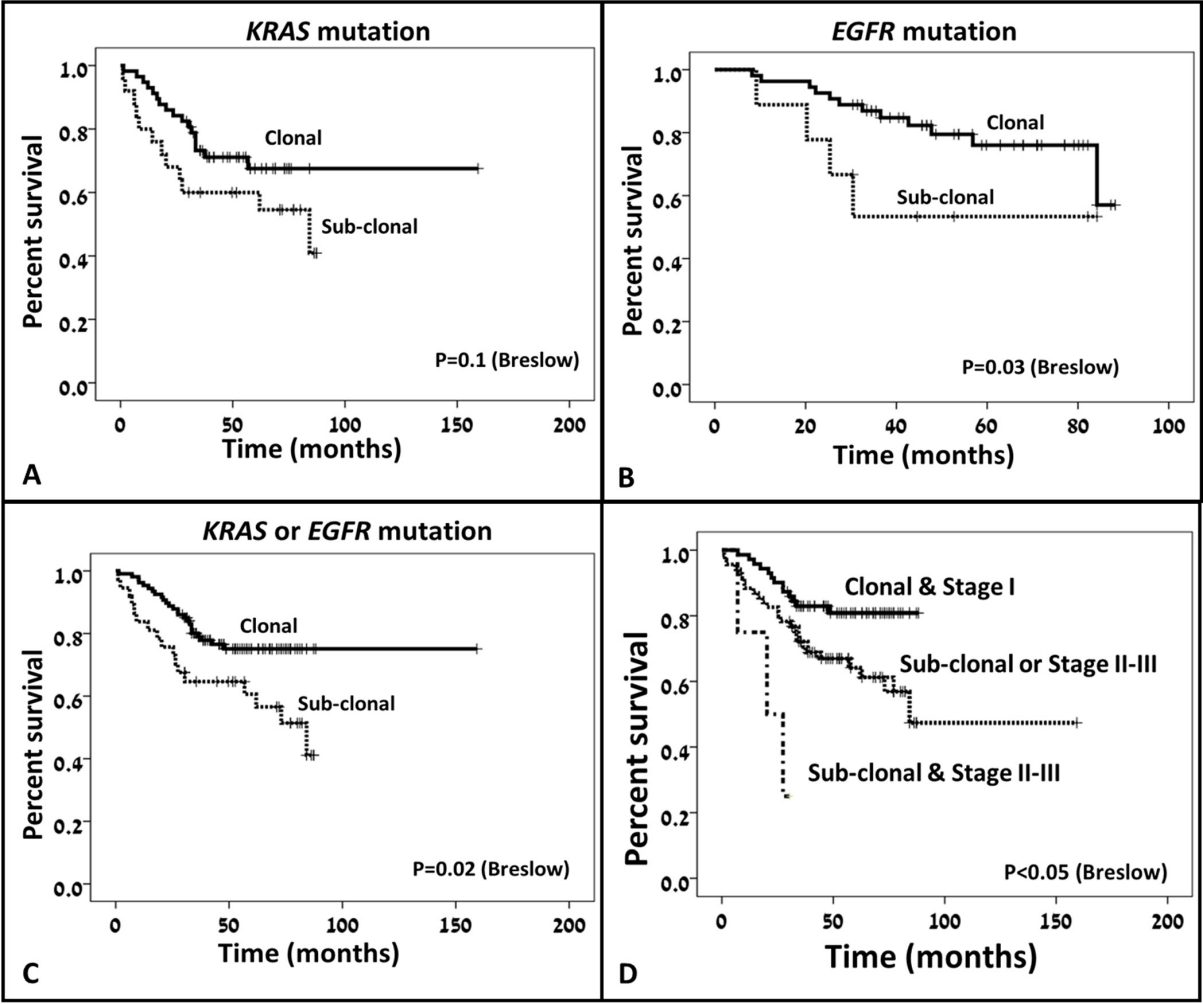
Clonality status effect on survival While sub-clonality for *KRAS* mutation showed only trend toward statistically significant association with reduced survival **(A)**, sub-clonality for *EGFR* mutation **(B)** and sub-clonality for either *KRAS* or *EGFR* mutations **(C)** were significantly associated with shorter survival times. A combined score including disease stage and clonality status could stratify the patients into 3 prognostic groups, where cases with clonal disease at stage I had the longer survival and cases with sub-clonal disease and more advanced disease stage had the shortest survival time **(D)**.

Another finding in our research is the association between sub-clonality and early disease stage. This finding stands in some contradiction to the association between sub-clonality and worse prognosis. This phenomenon can be potentially explained by understanding the different modes of evolution that can present as sub-clonality. Basically, the two major evolutionary models are linear evolution and branched evolution, and both can lead to sub-clonality [1]. In linear evolution model, if a sample is being analyzed before a new sub-clone eradicated the previous clone the result would be sub-clonality for the mutations carried by the new sub-clone. In a branched model, different sub-clones evolve in parallel leading to sub-clonality. We propose that the different types of sub-clonality are related to different associations with the clinical phenotype. Sub-clonality related to linear evolution would be expected in early disease stage and smaller lesions where nutrient and blood supply are abundant. This could explain the association reported between sub-clonality and early disease stage and smaller lesions [27, 28]. On the other hand, sub-clonality associated with branched evolution might be the type of sub-clonality that is associated with increased tumor fitness, development of treatment resistance and poor survival [9, 19, 20] (Figure 4).

Copy number gain of oncogenes including *EGFR*, *KRAS* and c-MYC has been reported in lung carcinoma, where it was associated with advanced stage and poor clinical outcome [42–45]. In the present study we did not find any association between mutant allele amplification and clinic-pathological variables. One potential explanation is that oncogene amplification is not restricted to the mutant allele copy and that amplifications could be present in the wild-type copy [42] thereby masking the clinical difference between the groups. Additionally, oncogene amplification pattern was reported to be heterogeneous within the tumor tissue [44] and it is possible that some cases with focal amplification were unidentified by our approach. Lastly, our sample set includes cases with early resectable disease, in which the associations between oncogene amplification and clinic-pathological variables might be less pronounced.

In conclusion, using a molecular morphometric approach we were able to demonstrate sub-clonality for *KRAS* and *EGFR* mutations in lung adenocarcinoma. We found that sub-clonality was associated with both early disease stage and with poor prognosis, suggesting that sub-clonality should not be regarded as a single entity, and could represent different evolutionary stages in tumor development.

## MATERIALS AND METHODS

### DNA extraction from formalin fixed paraffin embedded (FFPE) tissue samples

DNA extraction from 347 surgical specimens of lung adenocarcinomas, taken between 2007 and 2011, was performed as previously described [46]. Briefly, an area containing a high fraction of tumor cells was marked by a pathologist, microscopically dissected and DNA was extracted using the QuickExtract FFPE DNA Extraction kit (Epicentre, Madison, WI) according to manufacturer instructions. Following treatment with RNase A (Qiagen, Hilden, Germany), DNA was purified using the DNA Clean and Concentrator kit (Zymo Research, Orange, CA). The study was approved by the local ethics committee.

### Molecular morphometric approach

**Figure 5:**
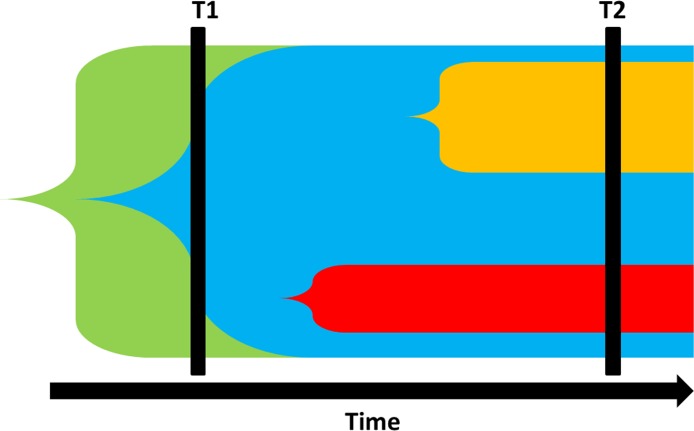
Tumor evolutionary scenarios associated with sub-clonality The horizontal axis represents time and the different colors represent different mutational events leading to new clones. Cancer driver mutations can be sub-clonal in both linear and branched evolutionary model. In linear evolution model (T1) sub-clonality can be identified it the tumor is sampled before the new mutation resulted in eradication of the ancestral clone (clonal sweep). Alternatively, in branched evolution different clones expand in parallel, also resulting in sub-clonality (T2). Depending on sampling time, sub-clonality can either represent early linear model associated with early stage disease or more advanced stage with different sub-clones associated with shorter survival times.

The tumor mass in solid tumors such as adenocarcinoma of the lung are composed of tumor cells as well as stromal, blood vessels and inflammatory cells. While the tumor cells carry the tumor promoting mutations, the non-tumor cells of the mass contain a wild-type copy of the gene. To determine the fraction of tumor cells carrying a specific gene mutation we used a dual approach, combining molecular and computerized morphometry tools. As detailed below, molecular tools including next generation sequencing were used to determine the relative number of mutated DNA copies in each sample. Additionally, the area from which DNA was extracted was scrutinized using computerized morphometry, to determine the fraction of tumor cells in each sample. Combining both results we were able to calculate the fraction of tumor cells carrying a mutation in each sample, or, in other words, the degree of sub-clonality of each sample with regard to the mutations examined (Figure 5).

### EGFR and KRAS mutation screening

*EGFR* mutation analysis was performed using the Cobas *EGFR* mutation test (RocheMolecular System Inc., Branchburg, NJ), that can detect mutations present in as little as 10% of the sample. Additionally, samples were screened for *KRAS* exon 2 mutations using high resolution melting technology [46], another sensitive method, that in our hands could detect mutation present in as little as 1% of the sample.

### Library generation and determining the fraction of mutated copies

In cases that screened positive for *KRAS* or *EGFR* mutation further analysis was performed to determine mutant allele fraction in each case. Toward this aim we used the Ion Torrent Personal Genome Machine (PGM) sequencer platform. DNA extracted from the tumor samples was PCR amplified using primers forward 5′-GGCCTGCTGAAAATGACTGAA-3′ and reverse 5′-GGTCCTGCACCAGTAATATGCA-3′ for *KRAS*, forward 5′- AGCATGTGGCACCATCTCAC -3′ and reverse 5′- AGACATGAGAAAAGGTGGGC -3′ for *EGFR* exon 19 and forward 5′- AATTCGGATGCAGAGCTTC -3′ and reverse 5′- GCATGGTATTCTTTCTCTTCCG -3′ for *EGFR* exon 21. Each primer pair was supplemented with Ion-Torrent adapters P1 and A, to allow binding to the Ion Sphere Particles (ISPs). Additionally, 20-30 different forward primers, each with a different barcode, were used for every genomic area amplified to allow the analysis of multiple samples in a single reaction. Amplicons were purified using the Qiagen PCR purification kit (Qiagen, Hilden, Germany) and were then sequenced using an Ion 314 chip and sequenced on the PGM for 65 cycles. We aimed for X1000 coverage to allow accurate determination of mutant allele fraction in each sample. Data from the PGM runs was initially processed using the Ion Torrent platform-specific pipeline software Torrent Suite v1.3.1 to generate sequence reads, trim adapter sequences, filter, and remove poor signal-profile reads. Generated sequence files were aligned to the genomic sequence of *KRAS* exon 2 and *EGFR* exons 19 and 21 and we determined the fraction of the mutation and the wild type copies of the gene in each sample using the Integrative Genomic Viewer (IGV 2.3) free software [47, 48].

### Establish standard curve to determine mutant allele fraction

To determine the mutant allele fraction for each case from the next generation sequencing results we built standard curves for point mutations and for deletions involving *KRAS* and *EGFR* mutation “hot-spots”. Toward this aim we synthesized gBlock DNA sequences ∼450bps long that include the mutation/deletion area. Additionally, the wild-type sequence was also synthesized. We then mixed wild-type and mutant sequences to generate synthetic samples with mutant allele frequency ranging between 10% and 90%. These samples underwent PCR amplification and next generation sequencing and the results served as standard curve for validation and standardization of the molecular results of the study.

### Quantitative image analysis

In order to establish the proportion of tumor versus non-tumor cells, histological slides retrieved from the archives of the pathology department were immunohistochemically stained with anti-cytokeratin antibody (Lab Vision, Fremont, CA) thus enhancing the observer's ability to visually separate between tumor cells and non-tumor elements (such as stroma, vessels and inflammation). For area measurements, stained slides were entirely scanned at a magnification of X20 using the dotSlide 2.0 virtual microscopy system (Olympus, Germany & Japan). The digital virtual images were loaded in an image analysis system (Image Pro Plus 6.3, MediaCybernetics, MA, USA). The total area of the tumor was segmented from other elements (stroma, lymphatic aggregates and normal colonic crypts) and measured. For cell number counts, representative images of the tumor and other tissue elements were further captured at a magnification of X400 and cells were counted using the Image Pro Plus program. The total number of cells within each tissue element (tumor, stroma, lymphocytes, normal) was then calculated by using a mathematical extrapolation. This information was used to calculate the fraction of tumor cells in the samples. To determine the reproducibility of the morphometric analysis the entire analysis process was re-performed on 12 cases. Bland-Altman analysis [49] was used to determine the agreement between repeated measurements and the percent difference was calculated.

### Determining KRAS and EGFR mutation sub-clonality

In order to determine the fraction of tumor cells carrying a specific gene mutation, we combined the data obtained from the molecular and morphometric analysis. Assuming that *KRAS* or *EGFR* mutations are present in one allele, the fraction of tumor cells carrying the mutation was calculated using the formula:

2*(mutant allele frequency)/(tumor cell fraction).

### Validation of amplification

To validate the findings in cases predicted to have *EGFR* mutant allele amplification based on the molecular morphometric approach we performed chromogenic *in-situ* hybridization (CISH) using the digoxigenylated ZytoDot SPEC *EGFR* Probe (CE-Marked), the ZytoDot pretreatment kit, and the ZytoDot CISH polymer detection kit, according to the supplier's protocol (Zytovision, Clinisciences, Montrouge, France). Briefly, the DNA probe and sections were denatured at 95°C and hybridized at 37°C overnight using a HYBrite instrument (Vysis, Downers Grove, IL).

### Statistical method

In order to identify the presence of sub-clonality in as little as 10% of the tissue samples, with a statistical power of 90% (beta 0.1) and alpha of 0.05, the calculated sample size needs to be 95 cases at least. Our cohort size meets the power analysis criteria.

Association between the presence of sub-clonality and patients' clinical and histological variables were tested using the Chi square test for categorical variables and Student T test or the Mann-Whitney U test for continuous parametric or non-parametric variables as needed. The impact of sub-clonality on patients' prognosis was calculated using the Kaplan Meier product limit method and the Log-Rank test for detecting significant differences between the groups. Two tailed p values of 0.05 or less were considered statistically significant.

## SUPPLEMENTARY MATERIALS FIGURES AND TABLES


