# Supplementary material for: Clinically significant sub-clonality for common drivers can be detected in 26% of KRAS/EGFR mutated lung adenocarcinomas
Source: Oncotarget. 2017 Apr 24;8(28):45736–49. doi: 10.18632/oncotarget.17399 (PMC5542222; doi:10.18632/oncotarget.17399)
Supplement: Supplementary file 1 [file oncotarget-08-45736-s001.pdf]

Clinically significant sub-clonality for common drivers can be detected in 26% of *KRAS/EGFR* mutated lung adenocarcinomas

SUPPLEMENTARY MATERIALS

Supplementary Table 1: Distribution of *KRAS* and *EGFR* mutation types

	Mutation	Number (%)
***KRAS* mutations**		
	c.34G>A	5 (5%)
	c.34G>C	3 (3%)
	c.34G>T	43 (40%)
	c.35G>A	25 (24%)
	c.35G>C	10 (9%)
	c.35G>T	13 (12%)
	c.37G>A	1 (1%)
	c.37G>T	4 (4%)
	c.38G>A	2 (2%)
***EGFR* mutations**		
	Exon 18	4 (5%)
	Exon 19	44 (50%)
	Exon 20	1 (1%)
	Exon 21	39 (44%)

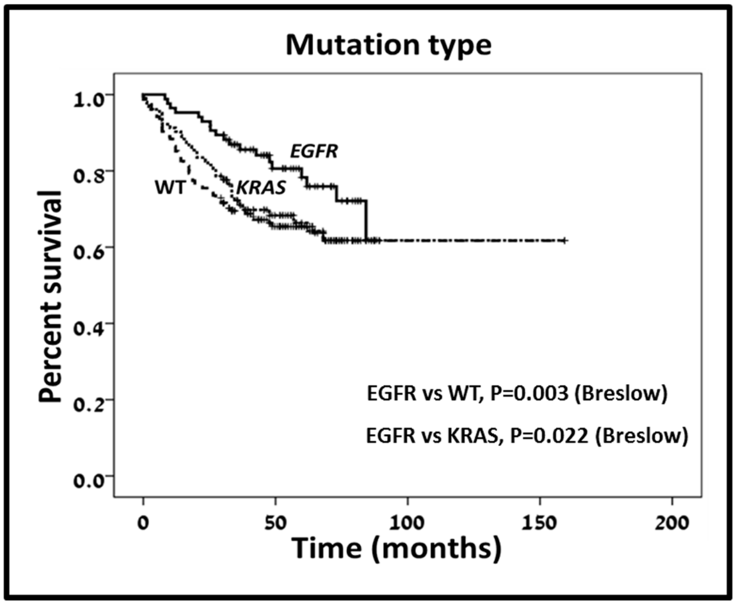

Supplementary Figure 1: Survival analysis Kaplan Meier curve. Survival analysis demonstrated that patients with tumors harboring *EGFR* mutation had significantly longer survival times compared to patients with tumors harboring *KRAS* mutation (P=0.022) or patients with tumors lacking *EGFR* or *KRAS* mutations (wild-type, p=0.003).
